# Giant Cell Arteritis: An Atypical Presentation Diagnosed with the Use of MRI Imaging

**DOI:** 10.1155/2016/8239549

**Published:** 2016-07-04

**Authors:** Siddesh Shambhu, Lisbet Suarez

**Affiliations:** Division of Academic Internal Medicine, Atlantic Health System, Overlook Hospital, Summit, NJ 07901, USA

## Abstract

Giant cell arteritis (GCA) is the most common primary systemic vasculitis in western countries in individuals over the age of 50. It is typically characterised by the granulomatous involvement of large and medium sized blood vessels branching of the aorta with particular tendencies for involving the extracranial branches of the carotid artery. Generally the diagnosis is straightforward when characteristic symptoms such as headache, jaw claudication, or other ischemic complications are present. Atypical presentations of GCA without “overt” cranial ischemic manifestations have become increasingly recognised but we report for the first time a case of GCA presenting as mild upper abdominal pain and generalized weakness in the context of hyponatremia as the presenting manifestation of vasculitis that was subsequently diagnosed by MRI scanning. This case adds to the literature and emphasises the importance of MRI in the evaluation of GCA patients without “classic” cranial ischemic symptoms.

## 1. Introduction

Giant cell arteritis is a chronic autoimmune vasculitis characterised by the infiltration of medium and large vessels by monocyte-derived giant cells leading to local and systemic inflammation. It is defined as a panarteritis that preferentially involves the extracranial branches of the carotid artery [1]. It has an estimated incidence of 20 cases per 1000 individuals and prevalence of 1 in 500 individuals [2]. Classic symptoms include temporal headache, jaw claudication, and fever but in around 40% of cases symptoms may be nonspecific that can delay prompt diagnosis [3].

The American College of Rheumatology (ACR) has established classification criteria to aid in diagnosis. To be deemed as having GCA, patients must meet 3 of the following 5 criteria: (1) age over 50; (2) new-onset localized headache; (3) temporal artery tenderness or reduced pulse; (4) ESR of 50 mm/h or higher; (5) abnormal temporal artery biopsy findings demonstrating mononuclear infiltration or granulomatous inflammation [4].

We present the case of a 79-year-old male who presented with generalized weakness and hyponatremia later revealed to be secondary to underlying GCA.

## 2. Case Report

A 79-year-old retired man with a past medical history of atrial fibrillation, cerebrovascular accident, hypertension, hypothyroidism, and myocardial Infarction presented to the hospital with a one-week history of generalized weakness and hyponatremia on routine blood work that had recently been ordered by his primary care physician.

On further questioning the patient admitted to having more shortness of breath recently with minimal exertion (walking 2 blocks) but there were no acute changes prior to admission. The patient denied any fevers, jaw pain, localized headache, or visual changes.

On physical examination vital signs including temperature were within normal limits with the patient noted to be in rate controlled atrial fibrillation and normal pulmonary examination. Abdominal examination revealed minimal left upper quadrant tenderness. Neurological exam was normal and there was no evidence of temporal tenderness. The examination of peripheral joints was normal.

His admission laboratory results revealed a hemoglobin of 12.1 and his sodium was 123 with normal renal and liver function tests.

Given the lack of any symptoms at this stage indicating a possible vasculitic or rheumatological cause for his presentation, the workup at this stage concentrated on looking for an underlying cause and correcting his hyponatremia. Serum osmolarity on admission was 269 msom/kg and urine osmolarity was 219 mosm/kg and urine sodium was 50 mEq/L giving the conclusion that the patient was hypotonic and hypovolemic. This was corrected with saline with dextrose.

A routine chest X-ray on admission revealed a 6 mm nodule and this provoked a CT scan of the thorax which revealed a few more nodules later deemed benign and possible aortic dissection. CT angiogram of the thorax revealed no dissection but possible aortitis of the upper abdominal aorta. Then the patient underwent CT angiogram of abdomen and pelvis which revealed atherosclerosis of the abdominal aorta but no evidence of aortitis.

**Figure 1:**
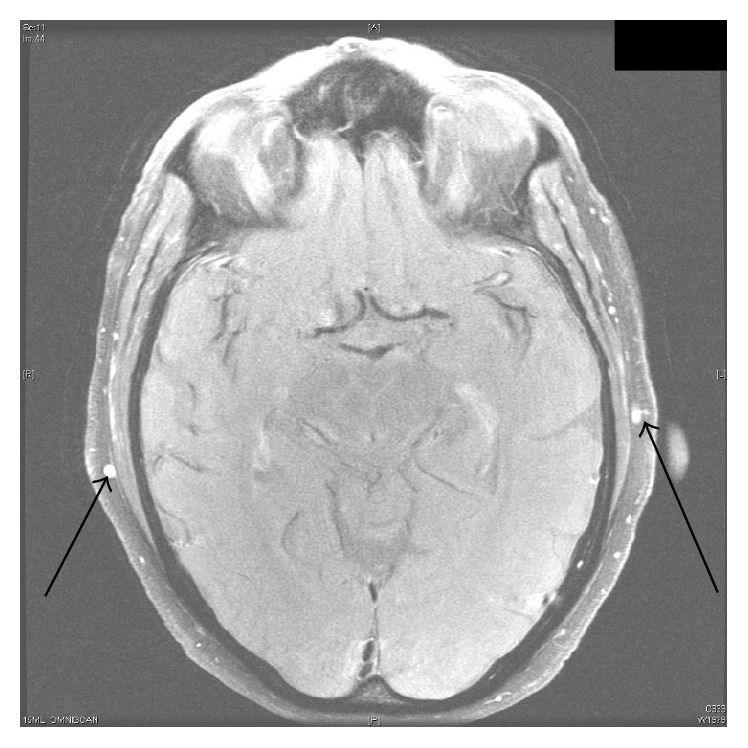
MRI showing mural thickening and enhancement of temporal arteries bilaterally (arrows).

**Figure 2:**
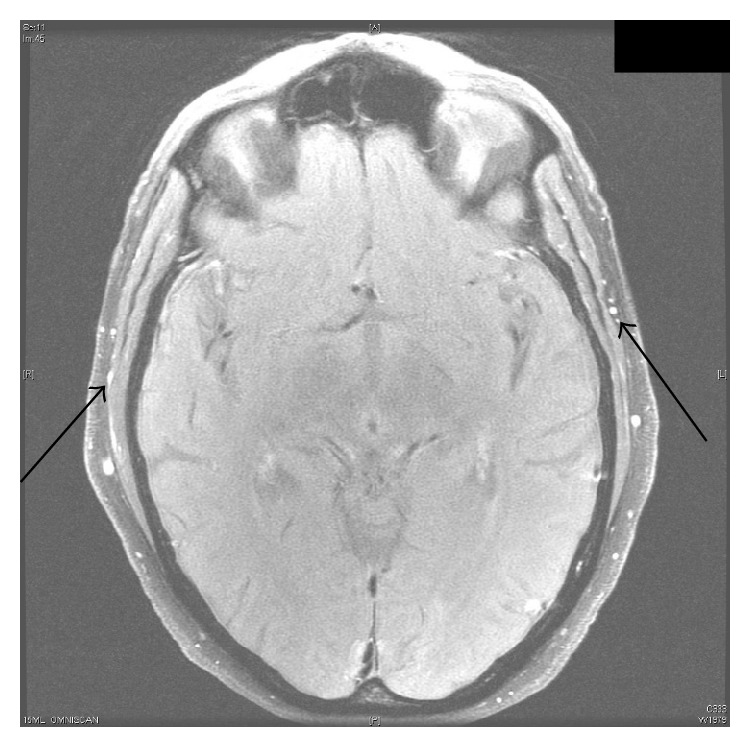
MRI showing mural thickening and enhancement of temporal artery “branches” bilaterally (arrows).

Given that the possibility of abdominal aortitis was previously raised on imaging a sedimentation rate was ordered which was raised (109) and the patient was worked up with ANA, ANCA, serum protein electrophoresis, and RPR but these all proved to be negative. As a result despite the patient having no typical features of GCA it was felt this should be excluded given the lack of any other possible explanation for his raised sedimentation rate. An initial ultrasound of his temporal arteries was ordered which was normal. The patient was reluctant to have any invasive biopsies done and also the need to hold the patient's warfarin for the procedure created additional risk for the patient. As a result MRI brain with GCA protocol was ordered with and without contrast and this showed mural wall thickening of bilateral superficial temporal and superficial occipital arteries indicative of GCA (Figures 1 and 2). The patient was commenced on prednisone 60 mg daily and following this introduction the patient felt constitutionally better over the next 72 hours and was subsequently discharged home on the current dose with rheumatological follow-up.

## 3. Discussion

The diagnosis of GCA is generally straightforward when characteristic symptoms such as headache, jaw claudication, or other ischemic complications are present. Atypical presentations of GCA without “overt” cranial ischemic manifestations have increasingly been reported however [5].

These have included symptoms such as dry cough [6], toothache [7], tongue pain, low back pain earache, hallucinations, and fever of unknown origin [8] and even symptoms of mononeuritis multiplex. It is unclear why GCA can provoke such a wide range of presenting symptoms but it is felt that some clinical subsets may involve unique pathologic pathways that are caused by differential expression of inflammatory cytokines [9].

We report a case of GCA presenting as mild upper abdominal pain and generalized weakness in the context of hyponatremia as the presenting manifestation of vasculitis. Diagnosis was made by MRI with GCA protocol. Recent studies have compared the use of MRI and the traditional modality of histological biopsy and found that the sensitivity and specificity of MRI for detecting GCA were 80.6% and 97.0%, respectively, when compared to biopsy (77.8%, 100%), respectively [10]. Ultrasound has also been proposed as a possible diagnostic modality but the technique is largely operator dependent and no large scale studies have analyzed its efficacy. In the case presented here the study was normal. Other diagnostic techniques include CT angiography and PET scanning but these are sparingly used given the widespread availability of other techniques.

The gold standard technique for diagnosing GCA has traditionally been biopsy of the temporal artery but as studies have shown there can be a marked false negative rate with this modality [11]. Generally this is due to biopsy missing the area of pathology given that GCA tends to have “skip” lesions and also that various forms of GCA exist that spare the temporal arteries and only involve the great vessels. Also naturally as with any surgical procedure there are inherent risks involved and indeed with the case described here the patient's use of warfarin for his atrial fibrillation and the risk of intraoperative bleeding made this choice even more unhelpful given the availability of alternative methods.

Treatment of GCA should generally be initiated immediately whenever the disease is strongly suspected. The general consensus is that a regime of 60 mg prednisone daily should be commenced and the treatment can last usually for several months. The dose is usually tapered depending on the patient's symptoms but a cautious approach is required to prevent relapse.

In conclusion the authors suggest that MRI with GCA protocol should be considered for all patients with suspected GCA particularly in those with atypical symptoms as in our patient to avoid delays in diagnosis.

## References

[1] Hunder G. G. (1997). Giant cell arteritis and polymyalgia rheumatica. *Medical Clinics of North America*.

[2] Evans J. M., O'Fallon W. M., Hunder G. G. (1995). Increased incidence of aortic aneurysm and dissection in giant cell (temporal) arteritis: a population-based study. *Annals of Internal Medicine*.

[3] Levine S. M., Hellmann D. B. (2002). Giant cell arteritis. *Current Opinion in Rheumatology*.

[4] Hunder G. G., Arend W. P., Bloch D. A. (1990). The American College of Rheumatology 1990 criteria for the classification of vasculitis. *Arthritis and Rheumatism*.

[5] Levin F., Schubert H. D., Merriam J. C., Blume R. S., Odel J. G. (2011). Occult temporal arteritis in a 54-year-old man. *Journal of Neuro-Ophthalmology*.

[6] Zenone T., Puget M. (2013). Dry cough is a frequent manifestation of giant cell arteritis. *Rheumatology International*.

[7] Hellmann D. B. (2002). Temporal arteritis: a cough, toothache, and tongue infarction. *The Journal of the American Medical Association*.

[8] Coeman M., De Pauw M. (2013). Large-vessel giant cell arteritis fever of unknown origin in a patient with a prosthetic valve. *Acta Cardiologica*.

[9] Weyand C. M., Goronzy J. J. (2013). Immune mechanisms in medium and large-vessel vasculitis. *Nature Reviews Rheumatology*.

[10] Bley T. A., Uhl M., Carew J. (2007). Diagnostic value of high-resolution MR imaging in giant cell arteritis. *American Journal of Neuroradiology*.

[11] Breuer G. S., Nesher G., Nesher R. (2009). Rate of discordant findings in bilateral temporal artery biopsy to diagnose giant cell arteritis. *The Journal of Rheumatology*.

